# 4-OI Attenuates Carbon Tetrachloride-Induced Hepatic Injury via Regulating Oxidative Stress and the Inflammatory Response

**DOI:** 10.3389/fphar.2021.651444

**Published:** 2021-05-25

**Authors:** Ruidong Li, Wenchang Yang, Yuping Yin, Xianxiong Ma, Peng Zhang, Kaixiong Tao

**Affiliations:** Department of Gastrointestinal Surgery, Union Hospital, Tongji Medical College, Huazhong University of Science and Technology, Wuhan, China

**Keywords:** carbon tetrachloride, 4-octyl itaconate, liver injury, NF-κB, oxidative stress, Nrf2

## Abstract

The liver is an important metabolic organ, and acute liver injury (ALI) is potentially lethal. Itaconate, a metabolic intermediate from the tricarboxylic acid cycle, showed emerging anti-oxidative and anti-inflammation properties, and an accumulating protective effect in multiple diseases, but its role in ALI still needs to be further explored. Here we established an ALI model induced by carbon tetrachloride in mice. Our results showed that 4-Octyl itaconate (OI), a derivate of itaconate, mitigated hepatic damage by improving liver function, reducing histopathological damage, and decreasing the death of hepatocytes. Additionally, OI decreased myeloperoxidase and thiobarbituric acid reactive substances (TBARS) levels in the ALI model. OI also inhibited the inflammatory response by reducing pro-inflammatory cytokine secretion (IL-6, TNF-α, IL-1β, and MCP-1) and infiltration of macrophages and neutrophils in the ALI model. However, administration of ML385, a specified Nrf2 inhibitor, eliminated the protective properties of OI in the CCl4-induced liver injury model by increasing hepatic damage and oxidative stress. Furthermore, OI increased the expression and nuclear translocation of Nrf2 and elevated the expression of heme oxygenase-1 and NAD(P)H quinone oxidoreductase 1, while knockdown of Nrf2 eliminated these effects in murine hepatocyte NCTC 1469 under CCl4 treatment. Moreover, we found that OI reduced serum High-mobility group box 1 (HMGB1) levels in CCl4-treated mice. Finally, OI inhibited nuclear translocation of factor-kappa B (NF-𝜅B) and inflammatory cytokine production in murine macrophages. In conclusion, these results indicated that OI ameliorated CCl4-induced ALI by mitigating oxidative stress and the inflammatory response. The possible mechanism was associated with the elevation of Nrf2 nuclear translocation and inhibition of HMGB1 mediated the nuclear translocation of NF-𝜅B.

## Introduction

The liver is a vulnerable organ, and numerous chemical agents can cause acute liver injury (ALI) (Hirao et al., 2020). ALI is a worldwide problem with high mortality rates. The mechanism of ALI is a complex interplay of inflammation, oxidative stress, and necrosis (AlSaad et al., 2020; Lyu et al., 2020). It frequently results from drugs, infections, and hepatic ischemia-reperfusion injury, among other causes (Du et al., 2019; Marra et al., 2020; Zhao et al., 2020). Carbon tetrachloride (CCl4) is well known to induce hepatic damage in murine ALI models, which mimic the hepatic chemical injury in humans (Lee et al., 2020). CCl4 is transformed into the highly reactive free radical trichloromethyl radical (•CCl3) and trichloromethyl peroxy radical (•OOCCl3) in the liver tissue by cytochrome p450, which leads to lipid peroxidation and cellar injury (Unsal et al., 2020). These free radicals also probably initiate the hepatic inflammatory response by activating macrophages to produce tumor necrosis factor-α (TNF-α) and other pro-inflammatory cytokines (Li X. et al., 2021). Hence, increasing the antioxidant pathway could represent a pivotal mechanism in protecting the liver during acute oxidative stress.

Metabolic remodeling of the tricarboxylic acid cycle is common during inflammatory macrophage activation. Itaconate was recently reported as a regulated mediator of the inflammatory response (Mills et al., 2018). Itaconate is synthesized by activating the immune response gene 1 (IRG1), which encodes cis-aconitate decarboxylase (CAD). Citrate, an intermediate metabolic product, is transformed to cis-aconitate by aconitate hydratase 2. Then cis-aconitate is catalyzed by CAD and generates itaconate (Li et al., 2020c). 4-Octyl itaconate (OI) is one of the cell-permeable forms of itaconate derivatives. OI exerts significant anti-inflammatory and anti-oxidative effects by activating the nuclear factor erythroid–related factor 2 (Nrf2) pathway (Tang et al., 2018). Nrf2 is a crucial transcriptional nuclear factor that regulates multiple anti-oxidative genes (Mallard et al., 2020). In normal conditions, Nrf2 is maintained at a low level by forming a complex with Kelch-like ECH-associated protein 1 (Keap1), by which Nrf2 is degraded via a ubiquitin-proteosome pathway (Kopacz et al., 2020). Some stimuli, such as oxidative stress, will dissociate Keap1 from Nrf2 by modifying cysteine residues in Keap1 and subsequently lead to the translocation of Nrf2 from the cytoplasm into the nucleus. In the nucleus, Nrf2 can bind with the cis-acting antioxidant response element (ARE) of multiple anti-oxidative genes such as heme oxygenase-1 (HO-1) and NAD(P)H: quinone oxidoreductase 1 (NQO1) and promote their expression, which generates cytoprotective effects (Wen et al., 2019). Administration of OI exhibited promising protective effects in various inflammatory and oxidative-related disease models (Li Y. et al., 2020). For example, OI treatment improved the survival rates and reduced inflammatory cytokine release in a lipopolysaccharide (LPS)-induced sepsis model (Mills et al., 2018). Moreover, OI showed a protective effect in hepatocytes in the liver ischemia-reperfusion damage model and exhibited anti-oxidative and protective properties in nonimmune cells (Yi Z. et al., 2020).

The high-mobility group box 1 (HMGB1) protein plays a crucial role in inflammation response. Normally, HMGB1 is contained intercellularly without the capability of causing inflammation. However, HMGB1 can be released extracellularly by immune cells during inflammatory stimulus or through necrotic and damaged cells (Khambu et al., 2019). Not surprisingly, HMBG1 is widely involved in a wide range of hepatic diseases, such as drug-induced liver injury, cholestasis, and liver cirrhosis (Lin et al., 2017; He et al., 2018; Khambu et al., 2019). Abnormal expression of HMGB1 in hepatic tissue is associated with CCl4-induced liver injury, and administration of a HMGB1-neutralizing antibody could protect hepatocytes by reducing the inflammatory response and oxidative stress (Chen et al., 2014). Extracellular HMBG1 interacts with many cell surface receptors, including the receptor for advanced glycation end products (RAGE) and toll-like receptor (TLR)-2,-,4,-9, and exerts pro-inflammatory effects via activating nuclear factor-κB (NF-κB) and mitogen-activated protein kinase (MAPK) pathways, which lead to the expression of inflammatory mediators including TNF-α, interleukin (IL)-1β, and IL-6 (Scaffidi et al., 2002; Tsung et al., 2005). NF-κB activation and excessive expression of these pro-inflammatory cytokines are involved in exacerbating hepatic damage (Liu et al., 2020).

In this study, we investigated the effect of OI pretreatment on CCl4-induced liver injury in a murine model. We revealed that OI attenuated CCl4-induced hepatic damage. The possible protective mechanism of OI is related to the elevation of Nrf2 nuclear translocation and reduced oxidative stress in hepatocytes. Moreover, OI concomitantly decreased serum HMGB1 levels in CCl4-treated mice. Administration of OI inhibited HMGB1-induced NF-κB nuclear translocation and inflammatory cytokines production in macrophages. All these findings highlight the clinical potential of OI as a treatment strategy for ALI.

## Materials and Methods

### Regents

OI and ML385 were obtained from Med Chem Express (United States). Carbon tetrachloride was obtained from Acmec biochemical (Shanghai, China). Alanine transaminase (ALT), aspartate transaminase (AST), myeloperoxidase (MPO), reduced glutathione/oxidized glutathione assay kit (GSH/GSSG) and superoxide dismutase (SOD) assay kit were purchased from Nanjing Jiancheng Institute of Biotechnology (Nanjing, China). Thiobarbituric acid reactive substances (TBARS) assay kit was obtained from Elabscience (Wuhan, China). 2,7-Dichlorofluorescein diacetate (DCFH-DA), 4’6-diamidino-2-phenylindole (DAPI) and BeyoECL Plus were bought from Beyotime Biotechnology (Shanghai, China). HMGB1 was purchased from HMGBiotech (Milano, Italy). Mouse TNF-α, IL-1β, IL-6, MCP-1 enzyme-linked immunosorbent assay (ELISA) kits were purchased from Dakewei Bioengineering (Shenzhen, China). HMGB1 ELISA kit was brought from Westang Biotech (Shanghai, China). The PrimeScript™ RT Master Mix, and TB Green Premix Ex Taq™ were obtained from Takara (Tokyo, Japan). Rabbit polyclonal antibodies against Nrf2, NF-𝜅B p65, phospho-NF-𝜅B p65, IκB-α, cleaved poly ADP ribose polymerase (PARP), F4/80 and cleavage Caspase-3 were purchased from cell signaling Technology (Beverly, MA, United States). Anti-heme oxygenase-1 (HO-1), anti-NADPH quinine oxidoreductase-1 (NQO-1), anti-lamin B1 and anti-glyceraldehyde 3-phosphate dehydrogenase (GAPDH) antibodies were brought from Proteintech (Wuhan, China). Fetal bovine serum and high glucose Dulbecco’s modified Eagle’s medium (DMEM) were brought from Gibco Life Technologies (Carlsbad, CA, United States). All other chemicals used were of the highest commercial grade.

### Animals and Experimental Procedures

All C57BL/6 mice (8–10 weeks old, weighted 24–26 g) were purchased from SPF Biotechnology Co., Ltd. (Beijing, China). Mice were kept in a specific pathogen-free and environmentally controlled room and with free access to standard laboratory food and water with 12-h light and dark cycle. All animal experiments were approved by the Animal Care and Use Committee of Tongji Medical College of Huazhong University of Science and Technology. OI was dissolved in saline with a 2 mg/ml concentration and was given via intraperitoneal injection (50 mg/kg body weight) 2 h before CCl4 treatment. ML385 was treated once a day for 2 days by intraperitoneal injection (30 mg/kg body weight). The acute hepatic injury was induced by intraperitoneal injection of CCl4 0.1 ml/kg (10 ml/kg bodyweight, CCl4/olive oil volume = 1:99). Generally, animals were divided into five groups (*n* = 5): 1) control group: mice were given same volume vehicle during the whole procedure; 2) OI group: mice received OI without CCl4 treatment; 3) CCl4 group: mice were treated with the same volume of saline vehicle and then were treated with CCl4; 4) CCl4 + OI group: OI was given 2 h before receiving the same dose of CCl4 given in the CCl4 group; 5) CCl4 + OI + ML385 group: ML385 was treated once a day for two days then OI and CCl4 were administrated as CCl4 + OI group. Twenty-four hours after CCl4 treatment, all animals were euthanized. Blood samples and liver tissues were collected.

### Assay of Serum ALT and AST

Commercial assay kits measured serum ALT and AST levels according to the manufacturer’s instructions (Nanjing Jiancheng Biological Technology. Nanjing, China).

### The Cell Culture and Treatment

The murine macrophage cell line RAW264.7 was obtained from the Cell Bank of the Chinese Academy of Science (Shanghai, China). The murine normal hepatic cell line NCTC1469 was purchased from China Center for Type Culture Collection (Wuhan, China). RAW264.7 and NCTC1469 cells were cultured in Dulbecco’s modified Eagle’s medium (DMEM high glucose) with 10% fetal bovine serum under a humidified atmosphere of 5% CO_2_ at 37°C. NCTC1469 and RAW264.7 cells were grown in six well plates (3*10^5^ per well) overnight. OI (final concentration: 125 μM) or same volume of vehicle was treated 2 h before CCl4 administration (0.5% v/v). Twenty-four hours after CCl4 treatment, NCTC1469 cells were harvested for further analysis. For RAW264.7 cells, OI (final concentration: 125 μM) or the same volume was added, and HMGB1 (final concentration: 2 μg/ml) was added 2 h later. Twenty-four hours after treatment, cells and supernatants were collected for the next analysis.

### Measurement of ROS Level

NCTC1469 cells were grown in six wells plates and treated as previously described in 2.4. Then cells were incubated with 10 uM DCFH-DA for 45 min. Images were captured and cells were harvested and recorded by FACSCanto II flow cytometer (BD Biosciences, San Jose, CA, United States). Data were analyzed using FCS express 3 (De Novo Software) and mean fluorescence intensity (MFI) was used to represented the ROS levels.

### Measurement of Oxidative Stress and Antioxidant Capacity in Hepatic Tissue

Each mouse liver was collected and washed using cold PBS and homogenized in PBS (weight/volume = 1:10). The homogenates were centrifuged at 4,000 g for 20 min and supernatants were collected. Then level of TBARS was evaluated by using commercial kit from Elabscience (Wuhan, China) according to provider’s instruction. The SOD activity and GSH/GSSG ratio were measured by using commercial kit from Nanjing Jiancheng Institute of Biotechnology (Nanjing, China) according to provider’s instruction.

### Polymerase Chain Reactions

Total RNA was extracted from cells or tissues by TRIzol reagent according to the manufacturer’s instructions. According to the manufacturer’s protocol, mRNA was reversely transcribed to cDNA using PrimeScript RT Master Mix (TAKARA, Japan). Then the expression levels of mRNA in each sample was measured by TB Green Premix Ex Taq according to the supplier’s protocol in the StepOnePlus system (Applied Biosystems, United States). All tests were run for 40 cycles consisting of denaturation at 95°C for 30°s, annealing at 60°C for 30°s, and an extension at 73°C for 30°s. The relative expression of each gene was evaluated using the 2^−ΔΔCt^ method, and *Gapdh* was used as the internal control. The primer pairs were used previously (Xiao et al., 2019; Zhang J. et al., 2020) and are listed below: *Tnf-α* F: 5-CCT​GTA​GCC​CAC​GTC​GTA​G-3, R: 5-GGG​AGT​AGA​CAA​GGT​ACA​ACC​C-3; *Il-6* F: 5-AGT​TGC​CTT​CTT​GGG​ACT​GA-3, R: 5-TCC​ACG​ATT​TCC​CAG​AGA​AC-3; *Il-1β* F: 5-GGG​CCT​CAA​AGG​AAA​GAA​TC-3; R: 5-TAC​CAG​TTG​GGG​AAC​TCT​GC-3; *Gapdh* F: 5-ATT​CAA​CGG​CAC​AGT​CAA​G-3, R: 5-CTT​CTG​GGT​GGC​AGT​GAT-3; *Mcp-1* F: 5-ACT​GAA​GCC​AGC​TCT​CTC​TTC​CTC-3, R: 5-TTC​CTT​CTT​GGG​GTC​AGC​ACA​GAC-3.

### Cytokine Activities by ELISA and MPO Activity

According to the manufacturer’s instructions, prospective enzyme-linked immunosorbent assay kits determined the levels of IL-6, TNF-α, IL-1β, and MCP-1 in serum and supernatant. The hepatic tissue MPO activity was assessed by a detection kit according to the manufacturer’s instructions.

### Terminal Deoxynucleotidyl Transferase-Mediated dUTP Nick-End Labeling (TUNEL) Assay

TUNEL was used to evaluate cell death in hepatic sections. Briefly, wax-embedded hepatic tissue sections were dewaxed and rehydrated using xylene and a graded series of ethanol. The sections were treated with 20 μl/ml proteinase K without DNase at room temperature for 30 min and washed with PBS 3 times. Then sections were incubated without light in a 50 μl TUNEL detection mixture for 1 h at 37°C. After washing with PBS three times, sections were observed under a fluorescence microscope. Five random fields (200X) were selected, and positive cells were measured by ImageJ software (National Institutes of Health, Bethesda, MD).

### Hepatic Histopathologic and Immunohistochemical Analysis

Hepatic tissues were fixed in 4% paraformaldehyde solution at 4°C for 24 h and embedded in paraffin. Then 4 μm thick sections were cut and stained with hematoxylin and eosin (H&E) according to standard procedure. Five random fields (200X) were selected and cellular damage was evaluated by standard morphologic criteria such as nuclear pyknosis and loss of architecture. The necrotic area rates were measured by ImageJ. Immunohistochemical analysis was performed in murine paraffin-embedded hepatic sections by incubated with Ly6G antibody (Servicebio Technology Co., Ltd., Wuhan, China) to detect inflammatory cells. Five random fields (200X) were selected and positive cells were measured by ImageJ software (National Institutes of Health, Bethesda, MD, United States).

### Immunofluorescence Analysis

RAW264.7 cells (2.5 × 10^4^/well) were grown on cover glass slips in 24 wells plate and treated as described previously. Then cells were washed and fixed in 4% paraformaldehyde for 20 min. Then cells were treated with 0.1% TritonX-100 for 10 min and blocked in 5% bull serum albumin for 60 min. NF-𝜅B p65 antibody (1:500) was added and incubated for 10 h at 4°C, and the second antibody was added and incubated in the dark for 60 min; then DAPI was incubated for 5 min to stain the nucleus. For F4/80 staining, briefly, liver tissue sections were dewaxed and rehydrated using xylene and a graded series of ethanol. After antigen retrieval, sections were blocked in 5% BSA for 60 min and then incubated with F4/80 antibody (1:200) overnight at 4°C. After washing with PBS three times, a second antibody was added and incubated in the dark for 60 min, then DAPI was incubated for 10 min to stain the nucleus.

### Annexin V/7-Amino-Actinomycin D Staining

The apoptosis in NCTC1469 cells were evaluated by APC Annexin V Apoptosis Detection Kit with 7-AAD (BioLegend, San Diego, United States) according to producer’s protocol. Briefly, stimulated cells were washed twice in cold PBS and resuspended in annexin V binding buffer. Add 5 ul APC marked annexin V and 5 ul 7-amino-actinomycin D (7-AAD) in 100 ul cells suspension and incubated in the dark for 20 min at room temperature. Add 400 ul annexin V binding buffer to each sample then analyzed by flow cytometry.

### RNA Interference

NCTC 1469 cells (40–50% confluent) were grown in a 6-well plate and transfected with 50 nM NRF2 specified siRNA or siRNA control using Lipofectamine 6,000 as we reported previously (Zhang et al., 2020b). The transfected cells were cultured 48 h after transfection and then used in the experiments.

### Western Blot Analysis

According to the manufacturer’s protocol, the proteins of hepatic tissues and cell lines were extracted using the cell lysis buffer for Western blot and IP (Beyotime Biotechnology). According to the manufacturer’s protocol, cell nucleus proteins were extracted using Nuclear and Cytoplasmic Protein Extraction Kit (Beyotime Biotechnology). The concentration of protein was determined by BCA Protein Assay Kit (Beyotime Biotechnology). Sodium dodecyl sulfate-polyacrylamide gel electrophoresis and immunoblotting were performed as we reported previously (Li et al., 2020a; Zhang et al., 2020b). Images were recorded with the Image Lab system and analyzed using ImageJ software.

### Statistical Analyses

The results were expressed as the mean ± standard deviation. Statistical analysis was performed by one-way analysis of variance and followed by Tukey’s multiple comparison test to evaluate significance. Analyses were performed using Prism8. *p* < 0.05 was considered significant.

## Results

### OI Mitigates CCl4-Induced Hepatic Damage

**FIGURE 1:**
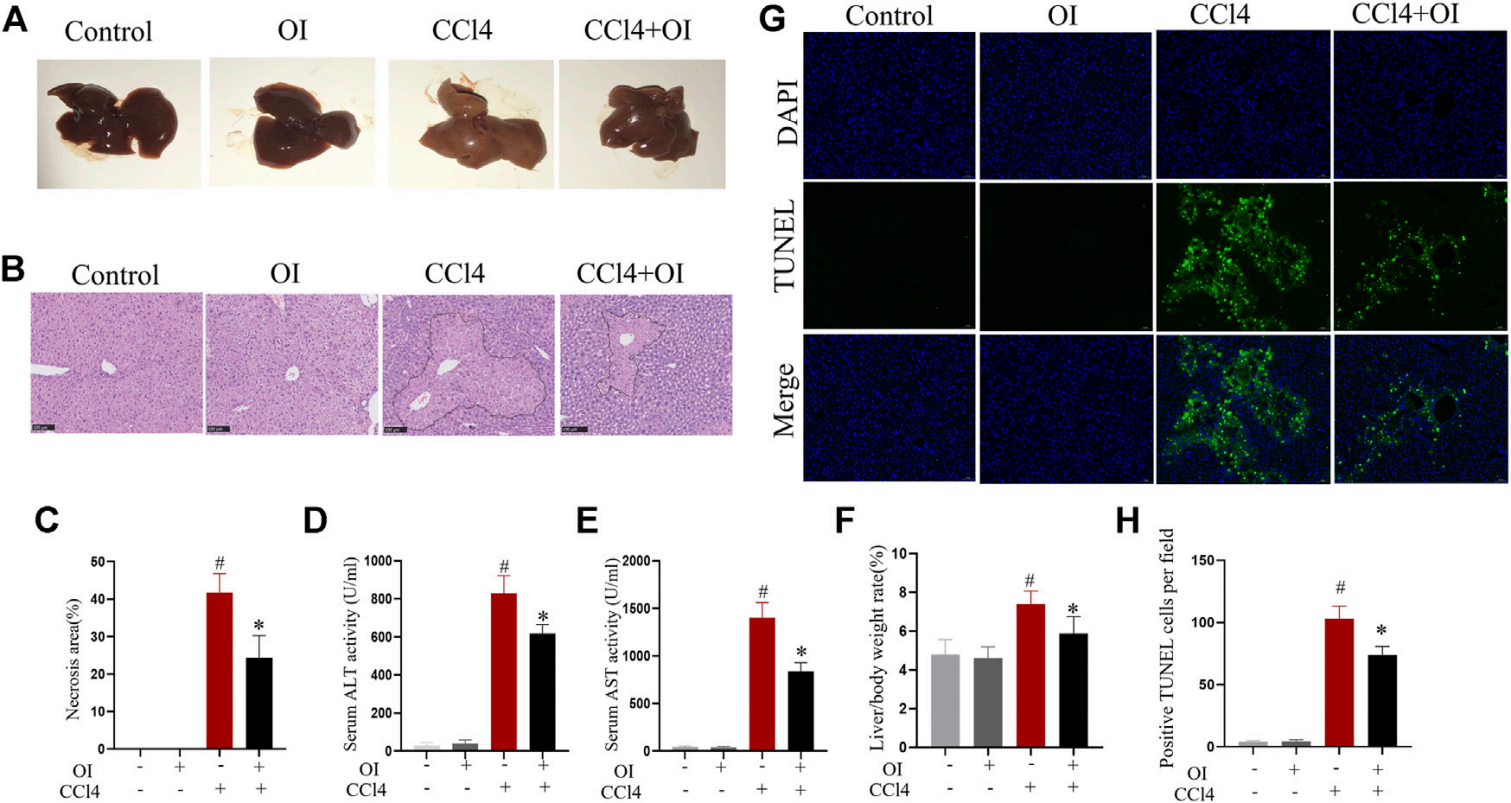
Effect of OI on CCl4-induced hepatic injury in mice. **(A)** Representative photographs of the whole liver were taken 24 h after CCl4 treatment. **(B)** Representative HE staining hepatic images of each group. The black border indicated the necrosis area. **(C)** The necrosis area was measured based on H&E hepatic sections from each group. **(D,E)** Serum ALT and AST levels of each group. **(F)** The liver/body weight of each group. **(G)** Representative TUNEL staining hepatic sections for necrotic cells in each group. **(H)** The quantification of positive TUNEL cells per field. Positive cells were counted in five visual fields per liver section from each group. Original magnification, ×200. Data are expressed as the mean ± SEM (*n* = 5 for each group); #*p* < 0.05 vs. control group; *p < 0.05 vs. CCl4-treated group.

As shown in Figure 1, the sections from single OI or control group mice showed no hepatocyte necrosis or cellular architecture damage and no abnormal hepatic function. In contrast, CCl4 induced hepatic damage in mice. In gross hepatic specimens, the liver’s appearance in the CCl4 group was markedly enlarged in size with a tight and smooth capsule, edema, lighter in color, and brittle texture. Administration of OI mitigated swelling and appearance of necrosis in the liver (Figure 1A). In the CCl4 group, H&E staining showed that CCl4-induced significant liver damage, including massive hepatocyte necrosis, loss of normal architecture, and an influx of inflammatory cells compared to the control group (Figures 1B,C). In contrast, mice that received OI showed a significantly decreasing necrotic area and histopathological damage (Figures 1B,C). Serum ALT and AST are two common indexes for evaluating the severity of hepatic injury. Our study found that ALT and AST significantly elevated in responses to CCl4 treatment. However, OI treatment markedly reduced ALT and AST activities compared to those mice that received vehicle treatment (Figures 1D,F). In addition, our results showed that the liver/body weight ratio significantly increased in the CCl4 group compared to the control group (Figure 1F), which was remarkably alleviated with OI pretreatment. Moreover, the TUNEL assay was used to evaluate OI’s effect on hepatocytes’ death in response to CCl4. As shown in Figures 1G,H, the apoptosis of hepatocytes significantly increased in the CCl4 group compared to the control group. However, pretreatment with OI markedly reduced the TUNEL-positive cells in the OI + CCl4 group compared to the CCl4 group. OI alone could not induce the death of hepatocytes.

### Effect of OI on the Infiltration of Inflammatory Cells in Mice with CCl4 Administration

**FIGURE 2:**
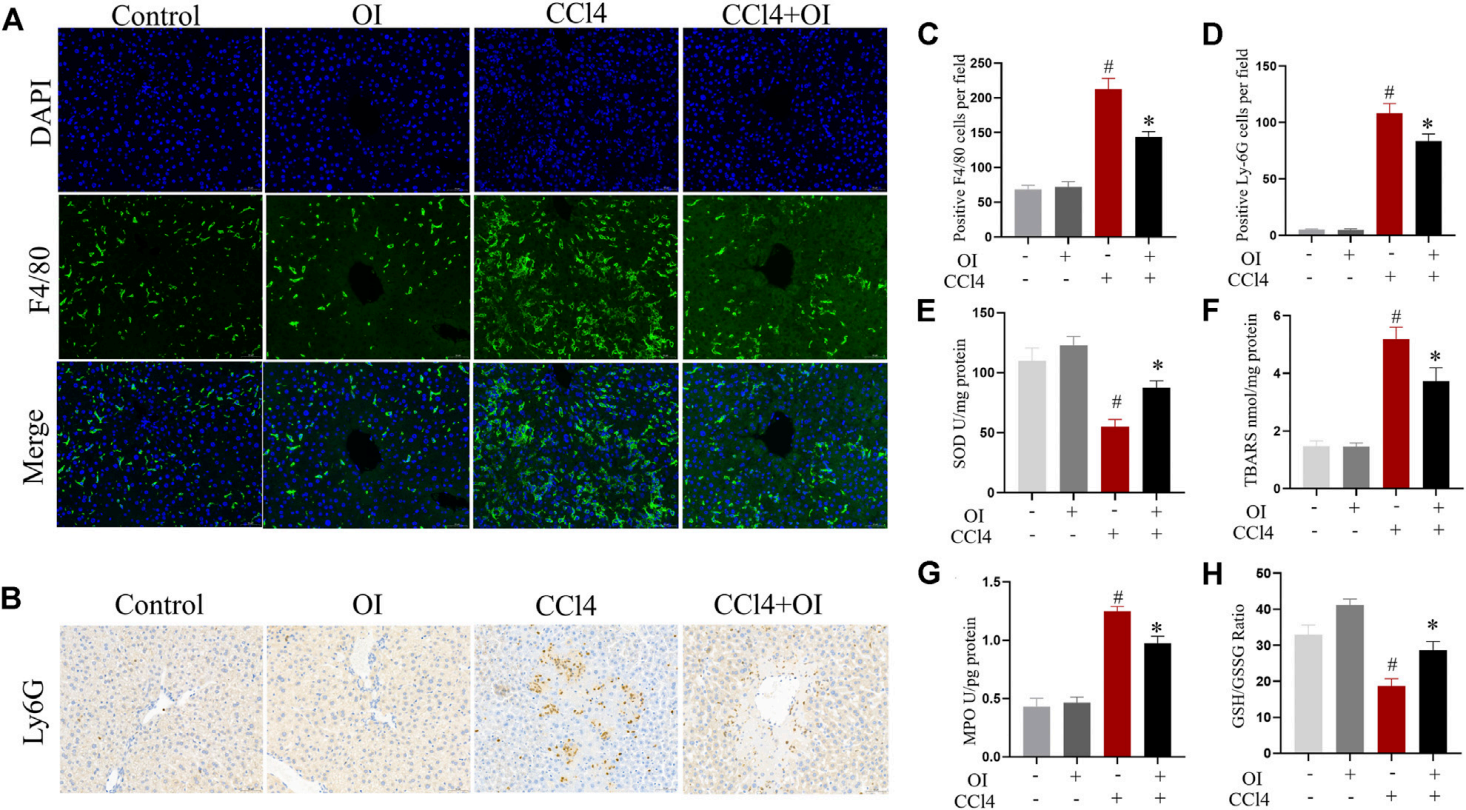
Effect of OI on the infiltration of innate immune cells and oxidative stress in mice. **(A)** Immunofluorescence detection of F4/80-positive cells in hepatic sections from each group; DAPI was used to show nucleus. **(B)** Immunohistochemical detection of Ly6G-positive cells in liver sections of each group. **(C)** Positive F4/80 cells per field were analyzed for five visual fields per liver sections from each group. **(D)** Positive Ly6G cells per field were analyzed in five visual fields per liver section from each group. **(E)** The activities of SOD were measured in each group. **(F)** TBARS levels were measured in each group. **(G)** Activities of MPO were evaluated in each group. **(H)** The GSH/GSSG ratio was measured in each group. Original magnification, ×200. Data are expressed as mean ± SEM (*n* = 5 for each group); #*p* < 0.05 vs. control group; *p < 0.05 vs. CCl4-treated group.

To evaluate OI’s effect in regulating inflammatory conditions in CCl4 treated mice, we analyzed inflammatory infiltrating cells such as macrophages and neutrophils with antibodies against F4/80 and Ly6G, respectively. Our study confirmed that F4/80-positive cells could be detected by immunostaining in hepatic tissues. Single OI treatment did not increase F4/80-positive cells infiltration compared to the control group. Notably, a massive increase of F4/80-positive cells infiltration was observed after treatment with CCl4. However, pretreatment with OI significantly reduced F4/80-positive cell recruitment (Figures 2A,C). Through immunohistochemistry, we detected infiltration of Ly6G-positive cells, which is considered a marker of neutrophils. Ly6G cells were rarely observed in the control and OI treatment groups. In the CCl4 group, a massive accumulation of Ly6G cells was observed in injured hepatic tissues. Moreover, the activity of MPO, which is considered a marker of neutrophils, was elevated in liver tissues by CCl4. (Figure 2G). However, OI administration significantly mitigated this neutrophil recruitment effect induced by CCl4 (Figures 2B,D,G).

### Effect of OI in Oxidative Stress in the Liver of CCl4-Treated Mice

The levels of SOD, TBARS, and GSH/GSSG ratio in the liver were measured to evaluate oxidative stress and antioxidant capacity in hepatic tissue. We found that CCl4 treatment could decrease SOD activity. However, OI could increase SOD activity (Figure 2E). Moreover, administration of CCl4 significantly increased the TBARS level (Figure 2F) compared to the control group. However, OI pretreatment led to a significant reduction of TBARS in the CCl4-induced hepatic injury model. The GSH/GSSG ratio is considered a pivotal index representing redox changes. In our study, administration of CCl4 could markedly reduce the GSH/GSSH ratio in hepatic tissues. In contrast, that reduction could be partially restored by OI pretreatment (Figure 2H).

### Effect of OI on the Production of Pro-Inflammatory Cytokines

**FIGURE 3:**
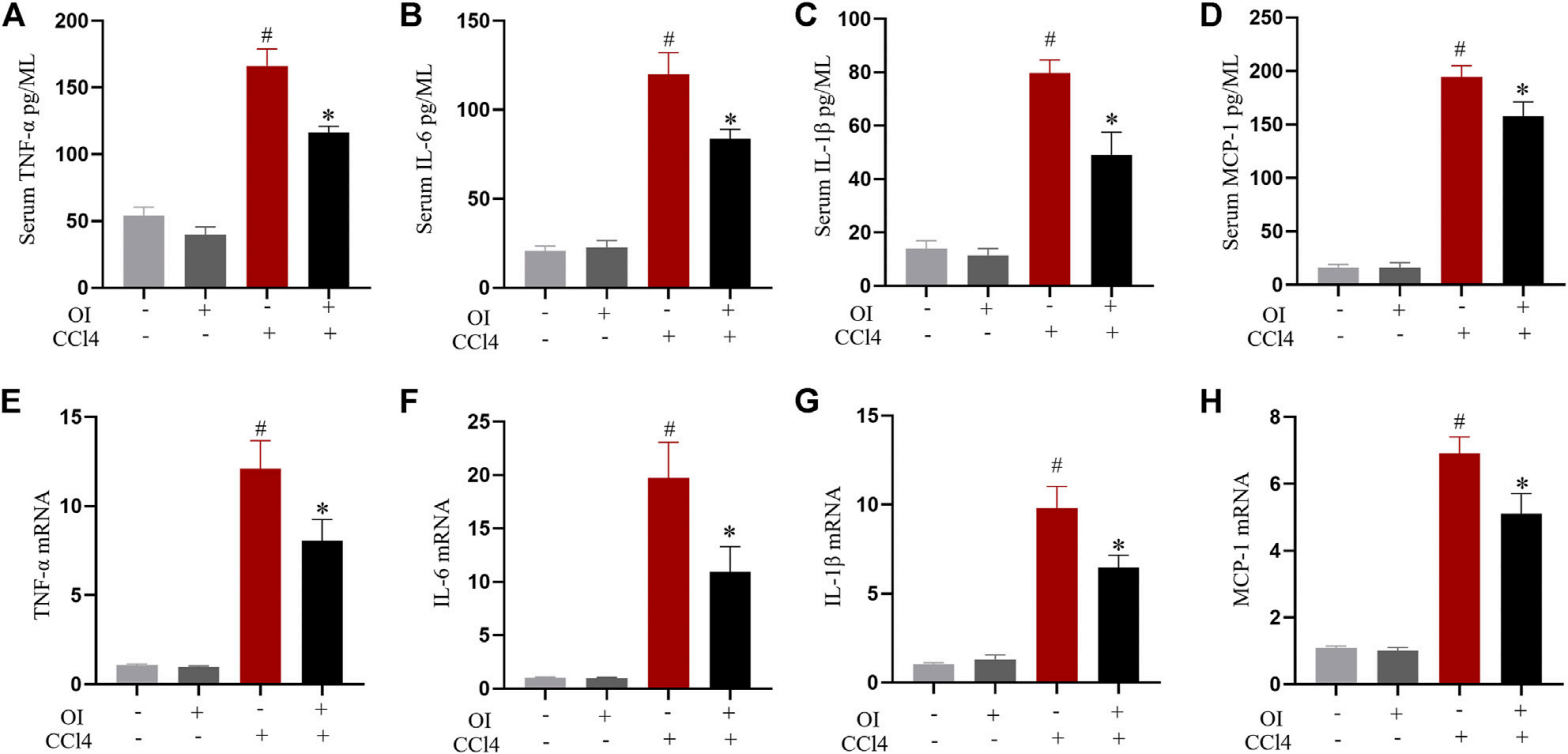
Effect of OI on serum cytokine levels and transcription of cytokines in liver tissues in mice. ELISA in each group measured serum levels of TNF-α **(A)**, IL-6 **(B)**, IL-1β **(C)**, and MCP-1 **(D)**. The mRNA levels of TNF-α **(E)**, IL-6 **(F)**, IL-1β **(G)**, and MCP-1 **(H)** in hepatic tissues were measured in each group. Data are expressed as mean ± SEM (*n* = 5 for each group); #*p* < 0.05 vs. control group; *p < 0.05 vs. CCl4-treated group.

Pro-inflammatory cytokines play an essential role during hepatic damage. Our experiment measured the transcription and serum levels of pro-inflammatory mediators such as TNF-α, IL-1β, IL-6, and MCP-1. Administration of CCl4 markedly increased the levels of serum TNF-α (Figure 3A), IL-1β (Figure 3B), IL-6 (Figure 3C), and MCP-1 (Figure 3D) compared to the control group, while OI pretreatment significantly attenuated CCl4-induced production of those pro-inflammatory cytokines. Similarly, the increase in the mRNA levels of those cytokines induced by CCl4 in liver tissues was inhibited by OI pretreatment (Figures 3E,F). And an OI single treatment showed no effect compared to the control group.

### OI Mitigates CCl4-Induced ROS Production and Death in Hepatocytes *in vitro*

**FIGURE 4:**
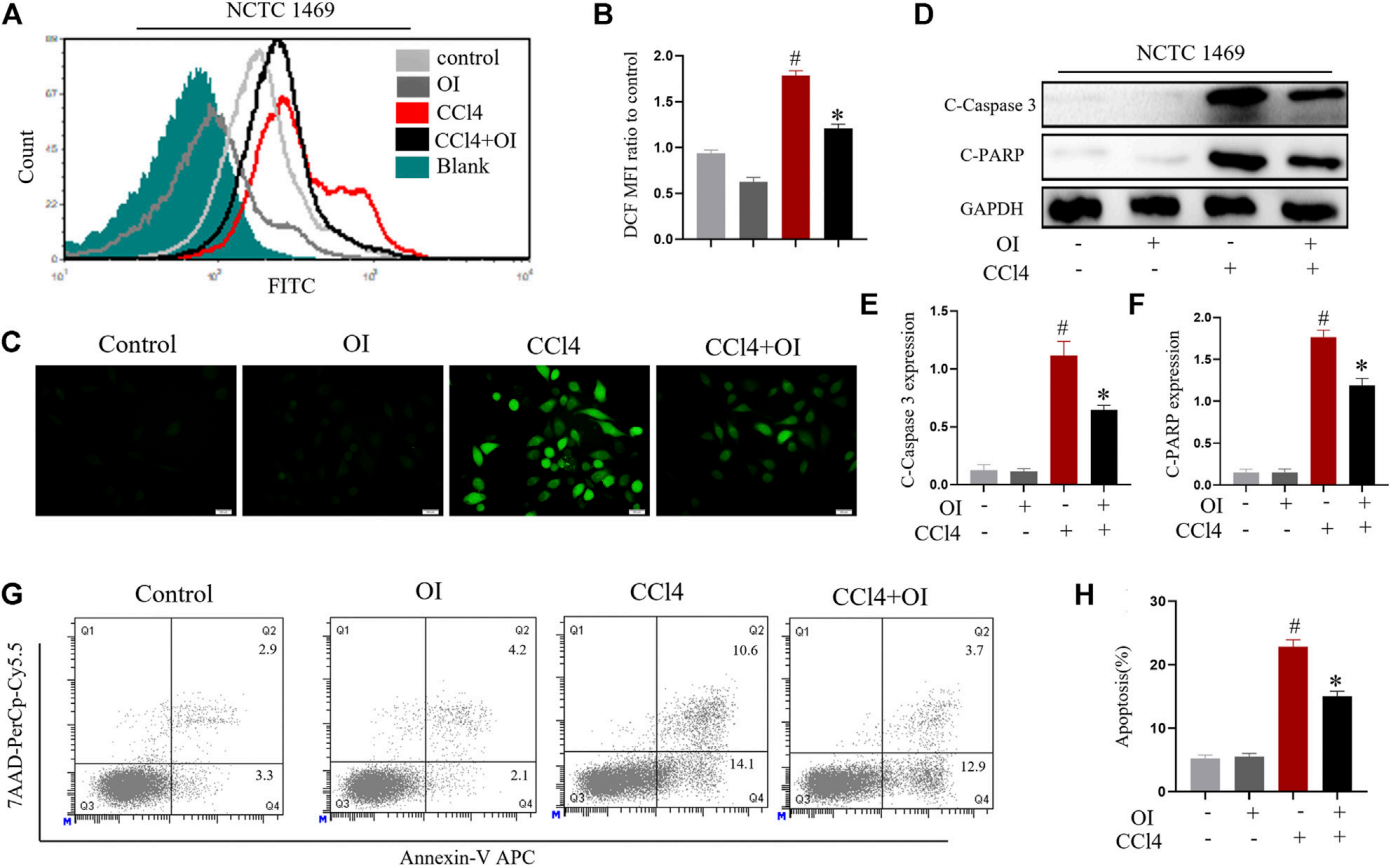
OI attenuated CCl4-induced cell death and ROS levels in NCTC 1469 cells. **(A)** Histogram of DCF FITC fluorescence. **(B)** Mean fluorescence intensity of DCF of each group. **(C)** NCTC 1469 hepatocytes were stained with DCFH-DA probe, and representative images were captured. **(D)** OI reduced increased levels of pro-apoptotic cleaved caspase-3 and cleaved PARP induced by CCl4 in each group, and we then performed an analysis **(E,F)**. **(G)** OI diminished the increased death of hepatocytes NCTC 1469 caused by CCl4, and we then performed an analysis **(H)**. *Gapdh* was used as an endogenous control. Original magnification, ×400. Data are expressed as mean ± SEM. All experiments were performed three times independently. #*p* < 0.05 vs. control group; *p < 0.05 vs. CCl4-treated group.

NCTC 1469, a murine normal hepatic cell line, was used to evaluate the effect of CCl4 in hepatocytes. ROS level was displayed using probe DCFH-DA. As shown in Figures 4A–C, CCl4 significantly enhanced ROS production in NCTC 1469 cells compared to the control group. In contrast, OI administration could markedly reduce ROS in CCl4 treated cells. We measured the respective cleavage form of caspase 3 and PARP, both of which are considered apoptosis markers. The expression of cleavage-caspase 3 and cleavage-PARP was markedly induced by CCl4 stimulation, while OI could significantly reduce their production during CCl4 treatment (Figures 4D–F). To further validate this observation, we used annexin V and 7AAD to measure the apoptosis rates in NCTC 1469 by flow cytometry. Similarly, the results showed that CCl4 markedly induced more hepatocytes’ death compared to the control cells, and OI treatment significantly reduced cell death and exerted a protective effect in CCl4-treated hepatic cells (Figures 4G,H).

### ML385 Eliminates the Protective Effect of OI in CCl4 Treated Mice

**FIGURE 5:**
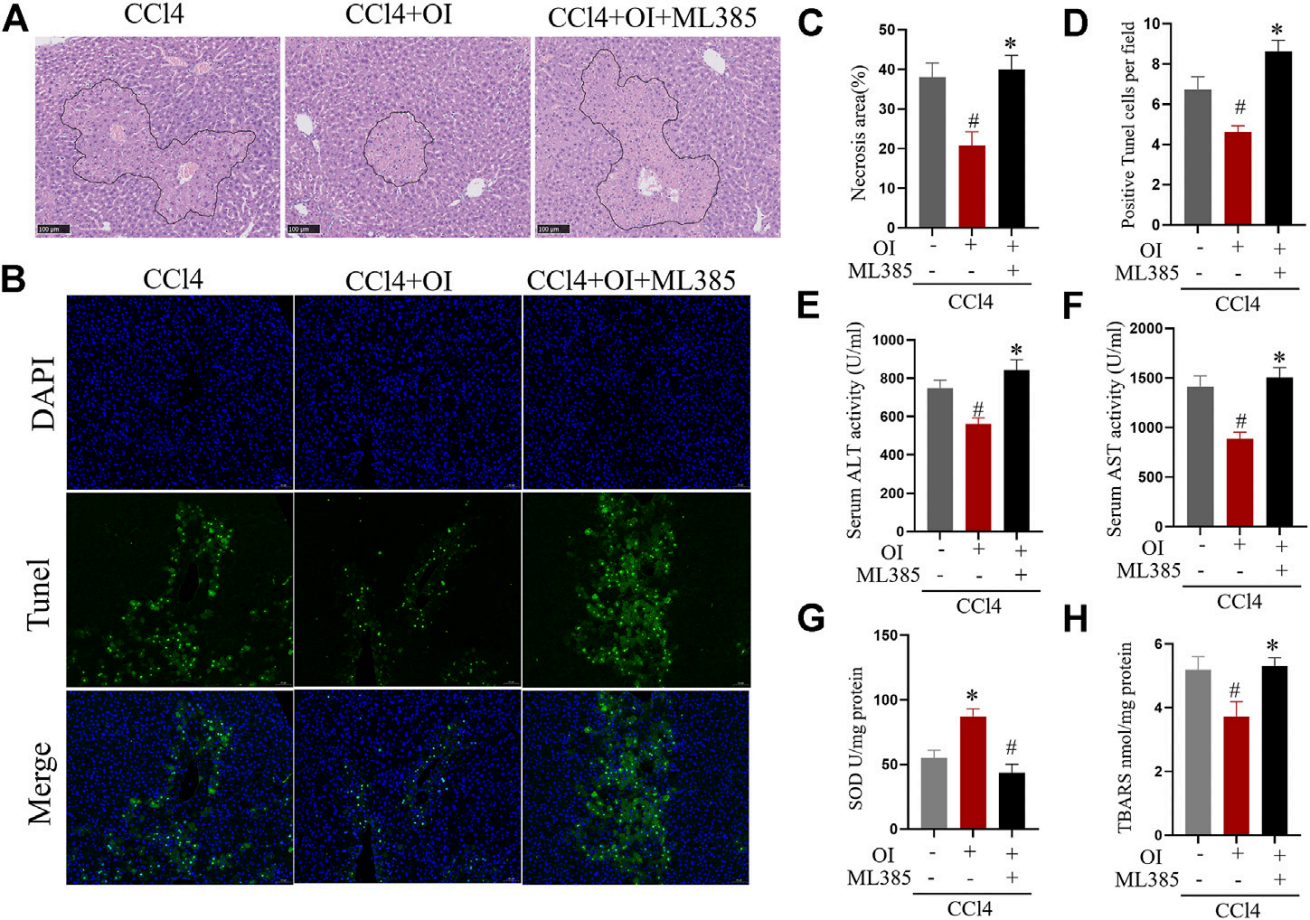
ML385 diminished the protective effects of OI in CCl4-induced liver injury in mice. Representative H&E staining images for the necrotic area **(A)** and TUNEL staining hepatic sections for necrotic cells **(B)** from the CCl4, OI + CCl4 OI + CCl4 + ML385 group. ML385 blocked the protective effect of OI. Next, the statistical analysis of necrotic area **(C)** and quantification of TUNEL-positive cells **(D)** were performed. Serum activities of ALT **(E)** and AST **(F)** were analyzed in CCl4, OI + CCl4, and OI + CCl4 + ML385 groups. SOD **(G)** activity and the level of TBARS **(H)** were analyzed in the CCl4, OI + CCl4, and OI + CCl4 + ML385 groups. Original magnification, ×200. Data were expressed as mean ± SEM (*n* = 5 for each group); #*p* < 0.05 vs. CCl4 group; *p < 0.05 vs. CCl4 + OI group.

To further explore OI’s protective mechanism in the CCl4-treated mouse model, ML385, a specific NRF2 inhibitor, was used to counteract OI’s effect. As shown in Figures 5A,C, CCl4-induced significant hepatic injury and OI administration mitigated liver histopathology as described before. However, pretreatment of ML385 significantly diminished the protective effect of OI in CCl4-induced injury in mice. In addition, we detected hepatocyte apoptosis in the liver by TUNEL assay. Similarly, CCl4 treatment-induced obvious cell death in liver tissues, which was markedly improved by OI pretreatment. However, this protective effect was eliminated by pretreating ML385, as shown in Figures 5B,D. Also, we found that the CCl4-induced elevation of ALT and AST was significantly inhibited by OI, while administration of ML385 to CCl4 + OI treated mice led to a significant increase in ALT and AST activities. In addition, TBARS level and SOD activity were measured to evaluate oxidative stress and antioxidant capacity, respectively. As shown in Figures 5G,H, OI pretreatment could decrease TBARS induced by CCl4 in hepatic tissues. However, the addition of ML385 eliminated the reduction of TBARS by OI pretreatment, and SOD activity was elevated by OI, while ML385 could block this effect in CCl4-treated mice.

### Knockdown of *Nrf2* Eliminates the Protective Effect of OI *in vitro*

**FIGURE 6:**
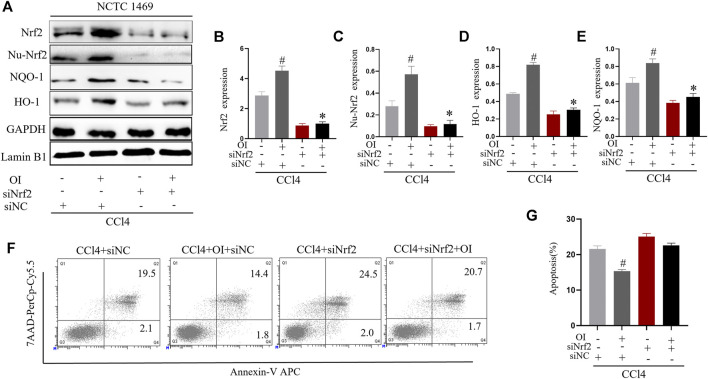
*Nrf2* knockdown attenuated the protective effect of OI in CCl4-treated NCTC 1469 cells. **(A)** Representative immunoblot images of Nrf2, Nu-Nrf2, HO-1, and NQO-1 in CCl4-treated NCTC 1469 cells with siRNA control (siNC) or siRNA *Nrf2* (siNrf2) treatment. **(B)** Immunoblot analysis of total Nrf2 expression in each group. **(C)** Immunoblot analysis of nucleus Nrf2 (Nu-Nrf2) expression in each group. **(D)** Immunoblot analysis of HO-1 expression in each group. **(E)** Immunoblot analysis of NQO-1 expression in each group. **(F)** The death of NCTC 1469 cells was measured by flow cytometry, and apoptosis rates was statistically analyzed **(G)** for each group. GAPDH and Lamin B1 were used as an endogenous control. Data are expressed as mean ± SEM. All experiments were performed three times independently. #*p* < 0.05 vs. siNC + CCl4 group; *p < 0.05 vs. OI + siNC + CCl4 group.

We used siRNA to knock down *Nrf2* expression in the murine normal hepatic cell line NCTC 1469 to explore the possible mechanism of OI’s protective effect in CCl4-induced hepatocyte damage. We successfully knocked down the expression of *Nrf2* in NCTC 1469 cells (Figures 6A,B). During the CCl4 administration process *in vitro*, the OI + CCl4 + siNC group significantly expressed more Nrf2 in the cytoplasm and nucleus than the CCl4 + siNC group. In CCl4 + siNrf2 and CCl4 + siNrf2 + OI groups, expression of Nrf2 was inhibited considerably (Figures 6A–C). In addition, we detected the expression of NQO-1 and HO-1 by immunoblotting. Similarly, the expression of NQO-1 and HO-1 was increased in the OI + CCl4 + siNC group compared to the CCl4 + siNC group (Figures 6A,C,D). However, NQO-1 and HO-1 were inhibited in CCl4 + siNrf2 and CCl4 + siNrf2 + OI groups (Figures 6A,C,D). To further explore the effect of OI and *Nrf2* knockdown in CCl4 treated hepatocytes, we analyzed apoptosis by flow cytometry. We found that the OI + CCl4 + siNC group showed markedly less cell death compared to the CCl4 + siNC group, while this effect was diminished in both the CCl4 + siNrf2 and CCl4 + siNrf2 + OI groups (Figures 6F,G).

### OI Reduces the CCl4-Induced Increased Serum Level of HMGB1 *in vivo*

**FIGURE 7:**
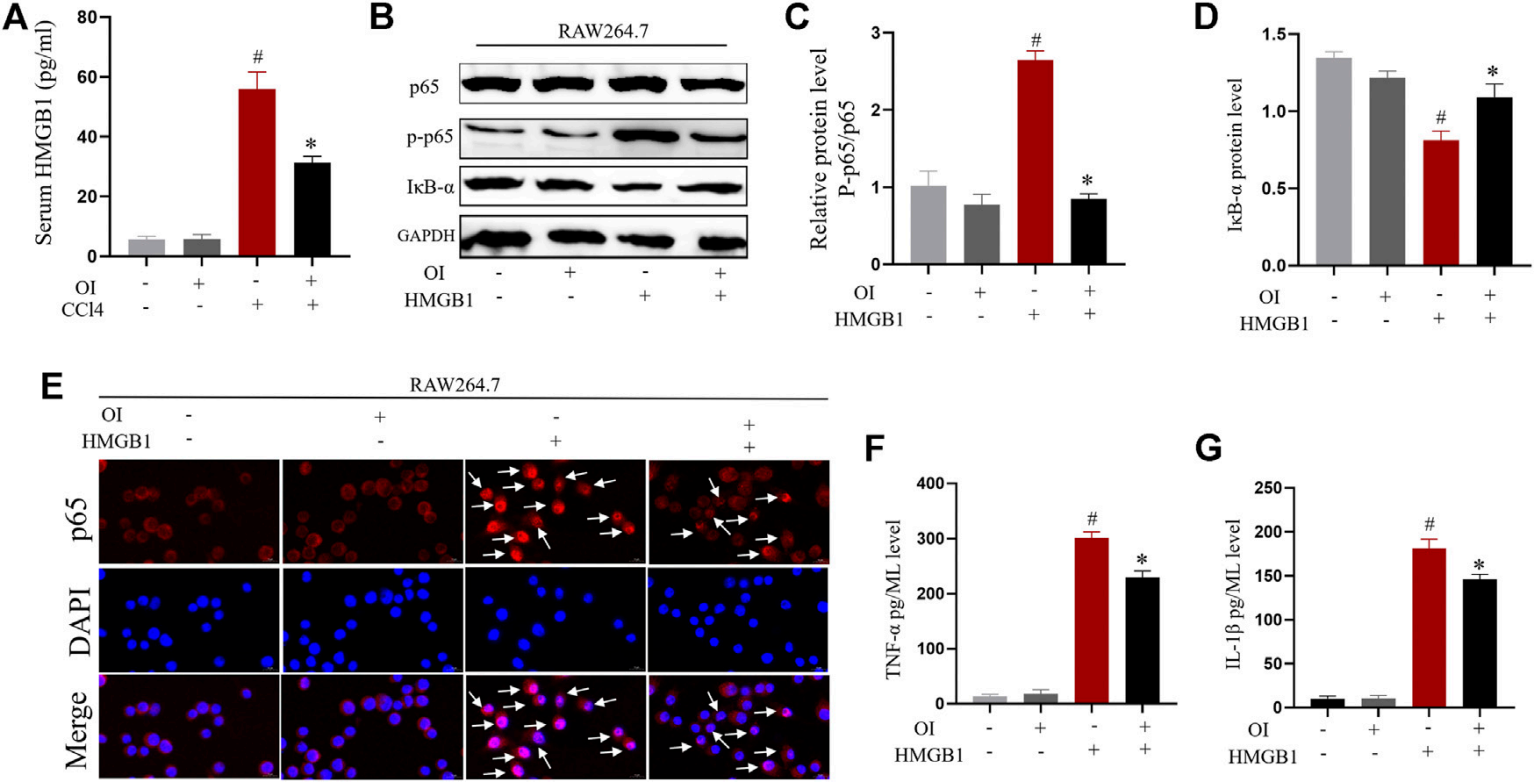
OI reduced HMGB1-induced activation of the NF-𝜅B pathway and inhibited pro-inflammatory cytokine synthesis in RAW 264.7 cells. **(A)** Serum levels of HMGB1 were analyzed in each group (*n* = 5). **(B)** Representative immunoblot images of NF-𝜅B p65, Phosphate-p65 (p-p65), and IκB-α for each group in RAW 264.7; *Gapdh* was used as an endogenous control. Analysis of the p-p65/p65 ratio **(C)** and IκB-α protein level **(D)** of each group. **(E)** Representative immunofluorescence detection nuclear translocation of p65 from each group in RAW 264.7 cells. Translocation of p65 is marked with a white arrow. The levels of pro-inflammatory cytokines such as TNF-α **(F)** and IL-1β **(G)** in RAW264.7 cell supernatant from each group were analyzed. Original magnification, ×400. Data are expressed as mean ± SEM. All experiments were performed three times independently. #*p* < 0.05 vs. control group; *p < 0.05 vs. CCl4 group.

It was reported previously that HMBG1 is associated with CCl4-induced liver injury (Chen et al., 2014). We sought to explore the impact of OI in CCl4-induced expression of HMGB1 in CCl4-treated mice. To measure HMGB1 release, ELISA analysis showed that serum HMGB1 was markedly increased by administration of CCl4 compared to the control group. However, pretreatment of OI in CCl4-treated mice significantly decreased HMGB1 in serum (Figure 7A), and OI alone did not induce HMGB1 release in serum.

### OI Inhibited Nuclear Translocation of NF-𝜅B and Production of Pro-Inflammatory Cytokines Induced by HMGB1 in Macrophages

Macrophage activation plays important roles in CCl4-induced hepatic injury. The above results indicated that CCl4 increased HMGB1 in serum. We verified that HMGB1 could enhance NF-𝜅B nuclear translocation by promoting phosphorylation of p65 and reducing the IκB-α level. At the same time, OI administration significantly inhibited this effect by decreasing the phosphorylation of p65 and restored IκB-α expression in RAW 264.7 cells (Figures 7B–D). Furthermore, nuclear translocation of NF-𝜅B p65 was detected by immunofluorescence. NF-𝜅B p65 nuclear translocation was increased by HMGB1, while OI administration inhibited such translocation (Figure 7E) in macrophages. This indicated that OI mitigated the NF-𝜅B nuclear translocation induced by HMGB1. The hallmark of macrophage activation is the secretion of specific pro-inflammatory cytokines. Therefore, we evaluated the activities of TNF-α and IL-1β in the supernatant. We found that the levels of TNF-α and IL-1β were significantly enhanced by HMGB1, while OI treatment reduced this effect (Figures 7F,G).

## Discussion

CCl4 can cause hepatic damage by increasing oxidative stress and the inflammatory response (Mohi-Ud-Din et al., 2019). Many chemicals showed a hepatic protective effect in the CCl4-induced mice model by activating the anti-oxidative pathway and increasing antioxidant synthesis (Ji et al., 2019; Yi R. et al., 2020; Lyu et al., 2020). A growing number of evidences have revealed that metabolic intermediates are involved in many physical and pathological processes, including the regulation of inflammatory response, oxidative stress, and cancers. As a metabolite from the tricarboxylic acid cycle, OI was recently found to have a strong anti-inflammatory property, regulating redox homeostasis, and showed a protective effect in many pathologies (Mills et al., 2018; Li Y. et al., 2020; Zhu et al., 2020). In a recent study, OI showed a protective effect in the liver ischemia-reperfusion injury mouse model by activating the Nrf2 pathway (Yi Z. et al., 2020). However, the protective effect of OI in CCl4-induced hepatic injury has not previously been investigated. Our present study supplied further evidence that OI could exert a protective effect in acute liver injury induced by CCl4 in mice by inhibiting the inflammatory response and attenuating oxidative stress. In addition, we provided evidence to support the hypothesis that enhancement of Nrf2 nuclear translocation in hepatocytes and inhibition of the HMGB1-induced NF-𝜅B p65 nuclear translocation in macrophages by OI might play a pivotal role in the alleviation of acute hepatic injury.

Hepatocyte damage leads to the release of ALT and AST from the cytoplasm into circulation. Serum ALT and AST are widely used to evaluate hepatic injury. In our study, CCl4 caused a hepatic injury, evidenced by inflation of mice liver, death of hepatocytes, and elevation of serum ALT and AST (Figure 1). However, OI treatment diminished ALT and AST serum levels and reduced hepatocytes death in liver tissues (Figure 1). In addition, it was reported that increasing infiltration of innate inflammatory immune cells such as macrophages and neutrophils was activated during the process of hepatocyte death (Antoniades et al., 2008). Macrophages and neutrophils are pivotal in the induction and regulation of inflammation (Cho and Szabo, 2020; Zhang et al., 2020a). Although these cells play important roles in eliminating pathogens and maintaining the inflammatory response at an early stage of inflammation, their overwhelming infiltration may exacerbate tissue damage and lead to an uncontrolled inflammatory response (Nakagaki et al., 2018). Our experiment used F4/80 and Ly6G antibodies to mark macrophages and neutrophils, respectively, in liver tissue. We demonstrated that the infiltration of macrophages and neutrophils increased by CCl4 treatment (Figures 2A,B). However, pretreatment of OI significantly reduced infiltration of macrophages and neutrophils in CCl4-treated mice (Figures 2A,B). Additionally, oxidative stress is another pivotal factor leading to hepatic damage. The mechanism of CCl4-induced liver injury is involved in increasing oxidative stress and impairment of the anti-oxidative defense system during the metabolism of CCl4 (Ramos-Tovar et al., 2018). Normally, an antioxidant defense system protects hepatocytes from injury by an oxidative stimulus. Nevertheless, cell damage occurs when cells encounter excessive oxidative stimulation that exceeds the capacity of the antioxidant defense system. The excessive oxidative stimulus will cause the oxidization of the cell's membrane and eventually destroy cells (Li et al., 2020b). It has been reported that the reduction of oxidative stress plays a pivotal role in diminishing hepatic injury. SOD is an enzyme that catalyzes the dismutation of the superoxide radical to oxygen and hydrogen peroxide. It can scavenge superoxide anion free radicals and protect cells from oxidative damage. In our study, the activity of SOD was diminished by CCl4 and restored by OI treatment (Figure 2E), which indicated that OI exerted a protective effect partially by alleviating oxidative stress. Meanwhile, MPO is an activation marker of neutrophils, the level of which represents the function and activity state of polymorphonuclear neutrophils (PMN). MPO can produce hypochlorite by hydrogen peroxide and chloride ion, and form free radicals. Therefore, MPO can induce oxidative stress and oxidative tissue damage when the excessive oxidants are generated by MPO (Li et al., 2016). In our study, the MPO level was increased by CCl4 administration, while OI pretreatment significantly diminished its level in CCl4-induced hepatic tissues (Figure 2G). Moreover, the TBARS level was decreased after OI treatment, indicating that lipid peroxidation was inhibited (Figure 2F). The GSH/GSSG ratio is an important index that represents oxidative stress in hepatic tissues, and increasing the GSH/GSSG ratio can help maintain immune function and detoxification (Muri and Kopf, 2020). We found that OI can increase the GSH/GSSG ratio in CCl4-treated mice (Figure 2H).

Excessive inflammation is another pivotal factor associated with CCl4-induced hepatic injury (Yu et al., 2014). Pro-inflammatory cytokines such as IL-6, TNF-α, IL-1β, and MCP-1 play crucial roles during the process of hepatic pathology induced by CCl4. It was reported that TNF-α and IL-1β are crucial in maintenance inflammation in CCl4-induced hepatic injury (Shin et al., 2013), and pretreatment with anti-TNF-α antibody was found to mitigate hepatic damage induced by CCl4 (Sato et al., 2014). Therefore, inhibiting the generation of excessive pro-inflammatory cytokines may alleviate hepatic inflammation and damage. Our results indicated that pretreatment with OI decreased the serum levels of IL-6, TNF-α, IL-1β, and MCP-1 and also inhibited the transcription of these cytokines in liver tissues induced by CCl4 (Figure 3). These findings indicated that OI protected mice from CCl4-induced liver damage by reducing the oxidative and inflammatory response *in vivo*. *In vitro*, similar results were observed. It was reported that CCl4 increased oxidative stress and led to cell death *in vitro* (Hamza et al., 2020). Here, we used CCl4 to treat NCTC 1469 cells, a murine normal hepatic cell line, and found that the ROS level and death of NCTC 1469 increased. However, OI could significantly diminish ROS accumulation and cell death induced by CCl4 *in vitro* (Figures 4A,C,G). Cleaved PARP and caspase 3 are important markers of cell apoptosis (Zhang et al., 2019). In our study, cleaved PARP and caspase 3 were evaluated by immunoblot, and we found that CCl4 elevated them in NCTC 1469 cells, while OI treatment could decrease their expression. *In vivo* and *in vitro* results indicated that OI exerted a protective effect possibly due to its anti-oxidative and anti-inflammatory properties.

It was recently reported that OI could activate the Nrf2 pathway by acetylating Keap1 (Mills et al., 2018). Activation of Nrf2 upregulated multiple downstream genes against oxidative stress and inflammation and exhibited remarkable protective effect in many disease models. For example, dimethyl itaconate protected doxorubicin induced by activating NRF2/HO-1 signaling (Shan et al., 2019). Itaconate protected mice from liposaccharide induced sepsis by activating the Nrf2 pathway, and this effect was eliminated in *Nrf2* knockout mice (Zhang S. et al., 2020). In our studies, we used a specific Nrf2 antagonist, ML385, to explore OI’s further protective mechanism in CCl4-induced liver injury. We found that the OI’s protective effect was diminished in the condition of ML385 pretreatment (Figure 5). In addition, we used specified siRNA to knockdown Nrf2 level in NCTC1469 murine hepatocytes. Transcription of HO-1 and NQO-1 is activated by Nrf2 (Tonelli et al., 2018; Alcaraz and Ferrandiz, 2020). Nrf2 signaling plays important roles in the anti-oxidative effect, and its activation shows effective protection in diseases. Nrf2-HO-1 pathway showed a protective effect against articular diseases by reducing oxidative stress and inflammation (Alcaraz and Ferrandiz, 2020). Activation of Nrf2-HO-1/NQO-1 was associated with neuroprotective effects (Wang et al., 2019). OI increased HO-1 and NQO-1 levels, and the CCl4-induced apoptosis effect in hepatocytes was inhibited by OI (Figures 6A,F). However, OI could not improve cell survival and increase HO-1 or NQO-1 levels in *Nrf2* null NCTN 1469 cells in the same condition (Figures 6A,F). Therefore, OI’s protective mechanism in CCl4-induced liver damage may be associated with increasing nuclear translocation of Nrf2 in hepatocytes.

Apart from the anti-oxidative property of OI, inhibition of inflammation is pivotal in alleviating liver injury. HMGB1 is a nuclear protein that can be translocated to the extracellular milieu under certain stimuli, such as necrosis and inflammation (Gou et al., 2020). HMGB1 plays important roles in inflammation diseases such as sepsis and acute liver injury (Chen et al., 2014; Gaskell et al., 2018; Deng et al., 2019). It was reported that serum HMGB1 was elevated in CCl4-induced mouse liver injury, and treatment with neutralizing antibody of HMGB1 could mitigate hepatic injury (Chen et al., 2014). The recruitment of macrophages is pivotal in removing death cell debris and pathogenic products. In addition, macrophages are important in the induction and development of inflammation by secretion of TNF-α, IL-1β, and other pro-inflammatory cytokines (Tacke, 2017; Guillot and Tacke, 2019). However, excessive activation of macrophages leads to an overwhelming inflammation response that can injure multiple organs. Elevation of HMGB1 could activate macrophages and enhance the inflammatory response, which exacerbated injury in multiple diseases (Andersson et al., 2000). We found that serum HMGB1 increased after CCl4 administration, while OI could reduce the serum HMGB1 level (Figure 7A). In addition, our results showed that HMGB1 could increase the phosphorylation of NF-𝜅B p65 and reduce IκB-α expression in macrophages (Figure 7B). HMGB1 could induce translocation of NF-𝜅B p65 into the nucleus (Figure 7E), which further increased pro-inflammatory cytokines expression (Figures 7F,G). NF-𝜅B nuclear translocation is critical in the secretion of inflammation cytokines (IL-1β and TNF-α) and plays important roles in hepatic damage (Meng et al., 2019; Zhang et al., 2020b). We found that OI could inhibit NF-𝜅B activation and production of IL-1β and TNF-α induced by HMGB1 in murine macrophages. Previous studies reported that oxidative stress could enhance inflammation through activation of NF-𝜅B (Nery-Flores et al., 2018; Sharma et al., 2018). In addition, OI can activate the Nrf2 pathway through acetylation of Keap1 (Mills et al., 2018). Furthermore, Nrf2 inhibits NF-𝜅B activation via enhancing antioxidant capacity and neutralizing ROS, thus reducing ROS-mediated NF-𝜅B activation (Soares et al., 2004; Sivandzade et al., 2019). Therefore, OI-modulated activation of NF-𝜅B may be due, at least in part, to its anti-oxidative potency via activating Nrf2. In conclusion, OI’s protective effect may be associated with a reduced inflammatory response through inhibition of NF-𝜅B activation in macrophages.

## Conclusion

We found that OI exhibited anti-oxidative and anti-inflammatory effects in CCl4-induced liver damage. Treatment of OI mitigated CCl4-induced hepatocytes death by reducing oxidative stress and the inflammation response *in vivo* and *in vitro*. The mechanism of this protective effect was due to two actions: 1) OI enhanced expression of Nrf2, HO-1, and NQO-1 against oxidative stress induced by CCl4 in hepatocytes; 2) OI inhibited CCl4-induced HMGB1 releasing and HMGB1 induced excessive inflammatory response in macrophages. Taken together, our findings indicate that administration of OI may be a potential therapeutic option for protecting against hepatic injury.

## Data Availability

The original contributions presented in the study are included in the article/Supplementary Material, further inquiries can be directed to the corresponding author.

## Abbreviation

ALI, acute liver injury; OI, 4-Octyl Itaconate; CCl4, carbon tetrachloride; HMGB1, High-mobility group box 1; ROS, reactive oxygen species; ALT, transaminase; AST, aspartate transaminase; SOD, Superoxide Dismutase; TBARS, Thiobarbituric acid reactive substances; PARP, poly ADP ribose polymerase; ELISA, enzyme-linked immunosorbent assay; MCP-1, monocyte chemotactic protein 1; IL-6, interleukin-6; IL-1β, interleukin-1β; TNF-α, Tumor necrosis factor-α; DCFH-DA, 2,7-Dichlorofluorescein diacetate; MFI, Mean fluorescence intensity; NRF2, Nuclear factor E2-related factor 2; NQO-1, NADPH quinine oxidoreductase-1; HO-1, heme oxygenase-1; NF-𝜅B, nuclear factor-kappaB; TUNEL, terminal dexynucleotidyl transferase (TdT) -mediated dUTP nick end labeling.
